# Rhythm and timing in laughter reveal that human vocal plasticity falls on a hominid continuum

**DOI:** 10.1038/s42003-026-10499-z

**Published:** 2026-06-25

**Authors:** Chiara De Gregorio, Marina Davila-Ross, Adriano R. Lameira

**Affiliations:** 1 ApeTank, Department of Psychology, University of Warwick, Coventry, UK; 2 School of Psychology, Sport and Health Sciences, University of Portsmouth, Portsmouth, UK

**Keywords:** Evolution, Zoology

## Abstract

Laughter is an important, universal form of human non-linguistic vocal expression and, being shared by all extant great apes, offers a valuable proxy for tracing the evolution of vocal control that ultimately enabled language. Yet surprisingly little is known about the evolution of its defining feature, rhythm. Here we show, through comparative analyses of laughter across all extant great apes (orangutans, gorillas, bonobos, chimpanzees, humans), that the laughter of the last common ancestor was already isochronous, becoming faster, more variable, and increasingly context-sensitive over hominid evolution. The evolution of laughter’s rhythm reveals a progressive increase in vocal rhythmic plasticity, with humans following the overall trajectory toward enhanced vocal control.

## Abstract (web summary)

Comparative analysis of laughter rhythm across all extant great apes reveals that isochrony is ancestral to hominids, with tempo, variability, and context-sensitivity increasing progressively toward humans, tracing a continuum of vocal plasticity.

## Introduction

Sound does not fossilize, making it difficult to trace the vocal origins of song, speech, and language. Comparative studies of the behavior of (nonhuman) great apes—our closest living relatives—provide the only extant model of extinct vocal capacities and adaptive functions among human ancestors. While all major branches of the Hominid family have evolved distinct call repertoires shaped by their species-specific socio-ecologies, one vocalization has been conserved across species and age–sex classes: laughter. Across great apes and humans, laughter occurs primarily during social play and other affiliative interactions, where it helps signal benign intent and maintain social coordination^1–3^. Shared by all great apes, including humans^4^, laughter offers a living record to phylogenetically contextualise and parse the changes in vocal control and rhythmic capacities that ultimately led to the emergence of speech and language among hominids. Because laughter in great apes and humans is inherently repetitive and cyclic—“ha-ha-ha-ha”—, variation in its temporal structure and organization may provide a window into evolutionary changes in respiratory–vocal coordination and motor timing across hominids. In particular, differences in the timing, regularity, and flexibility of laughter sequences could reflect broader evolutionary shifts in vocal control and rhythmic flexibility that distinguish genera within the Hominidae.

Earlier studies reported interspecific differences in laughter tempo limited to the tickling context and to a subset of great ape species^4^. In great apes, laughter is most reliably observed during playful interactions, particularly rough-and-tumble social play and tickling^4,5^. Building on these observations, here we present the first comparative analysis of laughter rhythm across all extant great apes, encompassing both social play and tickling contexts. Because communicative repertoires are shaped by species-specific socio-ecological conditions, including group size, social structure, and patterns of interaction^6,7^, variation in the temporal organization of vocalizations may emerge across species and social contexts. By comparing these different types of laughter, we reveal how tempo, its variability and regularity may have been shaped across species, social contexts, and along the phylogenetic tree. This integrative approach allows us to trace phylogenetic patterns in the temporal structure of laughter and explore how its rhythm may have evolved over 15 million years of hominid evolution, offering a window into the evolutionary refinement of vocal control.

We recorded laughter bursts from all five extant great ape taxa: four orangutans (*Pongo pygmaeus*), two gorillas (*Gorilla gorilla*), three bonobos (*Pan paniscus*), four chimpanzees (*Pan troglodytes*), and four humans (*Homo sapiens*), aged from six months to seven years old, most of whom were observed in ex situ settings. From these recordings, we annotated the starting point and duration of each call and extracted temporal features, including inter-onset intervals, their coefficients of variation, and rhythmic ratios. We used statistical modeling to investigate inter-onset interval duration (*t*_*k*_, measuring the interval from one sound onset to that of the following one - a proxy for laughter tempo) and their variability, accounting for both phylogenetic distance from humans and context (play or tickling). Additionally, we analyzed rhythmic ratios (the relative durations between consecutive *t*_*k*_) to assess whether laughter sequences across great apes were regular (isochronous) or variable. Assessing isochrony in laughter provides insight into the degree of vocal motor control and temporal regularity present across great apes, and into how such regularity may be modulated across different behavioral contexts.

## Results and discussion

**Fig. 1:**
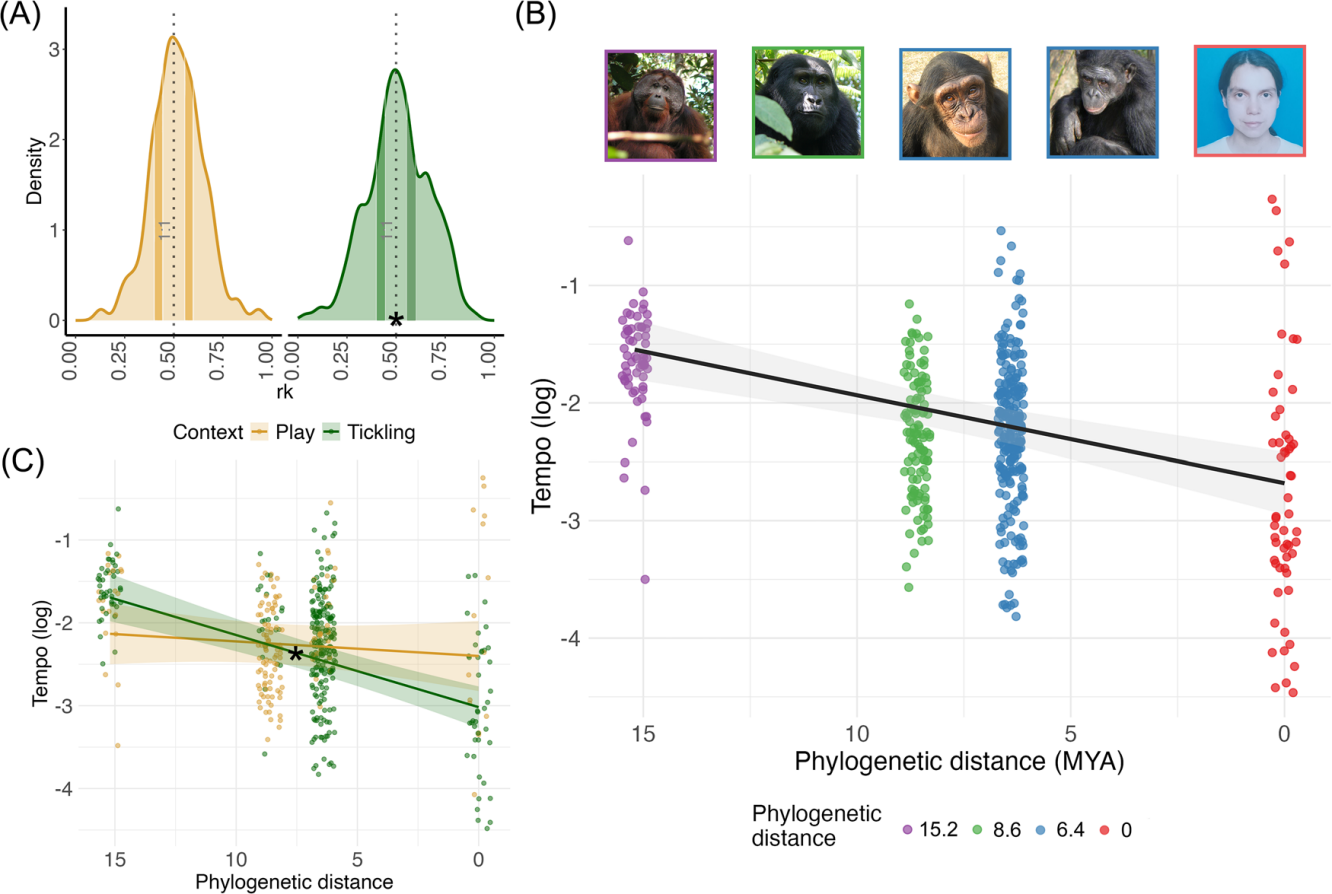
Evolution of temporal patterns of laughter in hominids. **A** Probability density function of rhythm ratios (*r*_*k*_) in the two behavioral contexts (play, in yellow, and tickling in green) derived from 140 laughter bouts across 17 individuals. White lines highlight on‑integer (0.440 < *r*_*k*_ < 0.555, lighter shade) and off‑integer (0.400 <*r*_k_ < 0.440 and 0.555 < *r*_*k*_ < 0.600, darker shade) ratio ranges. *Denotes *p* < 0.05, indicating a statistically significant correspondence between the empirical distribution and a small-integer rhythmic ratio category. **B** Variation in laughter tempo across species. Each dot represents an individual observation; color indicates phylogenetic distance (in million years ago, MYA). Each square contains an image of the corresponding species, with a matching dot color for intuitive reference. Credits to M. E. Hardus, M. Davila-Ross, E. Demuru. **C** Variation in laughter tempo across behavioral contexts (play, in yellow, and tickling in green). *Denotes *p* < 0.05. Sample sizes: *n* = 4 biologically independent animals for orangutans, *n* = 2 for gorillas, *n* = 3 for bonobos, *n* = 4 for chimpanzees, and *n* = 4 children.

Analyses showed that great apes’ laughter is isochronous, that is, involving regular timing between vocal bursts (GLMM_1_, *p* = 0.019, Supplementary Table 1). All taxa were included in the same model, with species as a random factor, to account for  interspecific differences. This result shows that great apes have been laughing in a recognizable way to modern humans for at least 15 million years, extending previous evidence of isochrony in orangutan vocalizations^8–10^ to other great apes, but also primates more generally (lemurs^11,12^, monkeys^13^, small apes^14,15^). However, isochrony was significantly dependent on context (GLMM_3_, *p* = 0.027; Supplementary Table 1, and Fig. 1A): tickling laughter was highly regular, while play laughter deviated significantly from strict regularity. This difference likely reflects the dynamic nature of social play, which involves the torsion, tensioning, thumping, and compressing of the thorax^16,17^, and with it, the disruption of a would-be regular respiratory cycle^18^. The “unaltered” rhythm of tickling laughter offers, thus, a stable window onto the evolutionary changes undergone by the phonatory-respiratory system of hominids. These changes may also relate to the progressive loosening of the coupling between vocal output and the respiratory cycle described in great ape laughter^4^.

In agreement with earlier work^4^, analyses also revealed that laughter rhythm accelerated along hominid evolution (LMM_1_, *p* < 0.001, Fig. 1B, and Supplementary Table 2), with species modeled along their phylogenetic distance. Notably, this trend was better captured by tickling laughter, than play laughter (LMM_2_, Play - *p* = 0.476; Tickling - *p* < 0.001; Supplementary Table 3, and Fig. 1C). Indeed, when breaking down laughter tempo by species, only humans modulated laughter tempo according to context (LMM_3_, Play vs Tickling; *p* < 0.014; Supplementary Table 3, and Supplementary Fig. S1), producing faster laughter during tickling than during play. Context-sensitive modulation was absent in great apes.

Phylogenetically-informed statistical analyses reveal a gradual shift toward greater temporal variability in laughter (LMM_4_, *p* = 0.004, Supplementary Table 3), with humans exhibiting the highest variability in laughter tempo (Supplementary Table 4), a result consistent with enhanced rhythmic range, a hallmark of advanced vocal control. Although this pattern emerges across the present dataset, the number of individuals per species remains limited, and future work with larger samples will help further refine species-level estimates of variability. In human laughter, variable timing is perceived as more socially and emotionally positive as opposed to rigid, stereotyped timing^19,20^, supporting the view that rhythmic functional span and temporal variability in laughter conveys socially relevant information about emotional state, intent, and disposition^12^ and that pronounced or unpredictable shifts in rhythm may amplify positive contagion^21,22^. Temporal variability in laughter decreased with phylogenetic distance from humans, reflecting an evolutionary gradual trend in vocal flexibility and its social functions across the Hominid family.

By revealing both conserved and derived rhythmic features, our results chart an evolutionary pathway toward increased vocal flexibility in a behavior retained across millions of years. These findings provide rare empirical evidence for a shift toward faster, more variable, and more context-sensitive rhythms in humans, a trait that likely paved the way for the emergence of speech and language. Laughter, in this view, is not merely a social signal but an accessible model for understanding the deep evolutionary roots of human vocal communication.

## Methods

### Subjects and recordings

Audio recordings were collected from four species of non-human primates (four orangutans, two gorillas, three bonobos, and four chimpanzees) at seven different institutions between 2004 and 2006. Young non-human great apes are relatively rare in captive populations, which limits sample sizes for this age class. Additionally, we collected recordings of laughter from four kids in their private homes. Details of ID, sex, age, and location of recording can be found in Supplementary Table 5. All subjects were acoustically recorded in their home environments during controlled, playful interactions with familiar humans, who elicited both play and tickle-induced vocalizations. Most of the tickling recordings from non-human apes were previously included in Davila-Ross and colleagues^4^, and we re-analyzed these original data points for the current study. This procedure has been reliably employed to investigate spontaneous vocal behavior across multiple species^23^. Recordings were made using a Sony WM-D6C with a Sennheiser ME 60 microphone and a Nagra IV-SJ with a Sennheiser MKH 816 microphone. We complied with all relevant ethical regulations for animal use at all zoological facilities where the recordings were collected. At the time the recordings were obtained, formal ethical approval was not required for non-invasive observational research under the guidelines of University of Veterinary Medicine Hannover, where MDR was then affiliated. The human participants were infants recorded during natural play interactions with their mothers in their homes. At the time of data collection (over 20 years ago), oral consent was obtained from the mothers. All ethical regulations relevant to human research participants were followed.

### Statistical analyses

Audio files in WAV format were converted to ESPS and resampled to 22,050 Hz. A 60 Hz high-pass filter was applied to reduce interference from electrical noise. Recordings with a signal-to-noise difference below 2 dB were discarded to increase the quality of the recordings. If noise masked the recordings with random fluctuations or if the onset/offset of the calls were unclear, these recordings were not included in the study. While it needs to be noted that the recordings were not perfectly clean from noise, which is to be expected from the majority of such acoustic studies, the impact of such factors is smaller if the focus is on temporal patterns, like in this study. Start times were aligned to 0.0 s, and direct current offsets introduced by tape recording were corrected to center the waveform on the zero-voltage axis and to remove low-frequency recording artifacts. Amplitudes were normalized to the full scale. All processing was performed with X-Waves 5.3 (Entropic Research Lab, Washington, DC). We annotated the starting point and duration of each call, and whether it belonged to the same bout of the preceding call or not. A call was defined as a continuous sound element without a sound gap. Following^4^, consecutive calls belonged to the same bout if the interval duration was less than 8 milliseconds or if they had the same mode. Two bouts belonged to the same series if they were separated by an interval of less than 1 second. We focused on bouts that had at least three calls, and selected a total of 140 bouts, of which 42 were laughter bouts from bonobos, 35 from chimpanzees, 34 from gorillas, 13 from humans, and 16 from orangutans. We used the software R^24^ to calculate the duration of the intervals between the starting point of a call within a bout and that of the following one belonging to the same bout (hereafter *t*_*k*_, following^11^), obtaining 458 *t*_*k*_ values (bonobos 112, chimpanzees 116, gorillas 112, humans 56, and orangutans 62).

We performed all statistical analyses using the software R^24^. Prior to model fitting, we created a new variable that is a factor encoding the phylogenetic distance of each species from humans based on ref. ^25^. To test the validity of our modeling approach, we compared our full models with their respective null models (containing random factors only) and proceeded with a post-hoc test when full and null significantly differed (Anova with chi-sq argument^26^).

### Laughter tempo

We fitted a first linear mixed-effects model (*Lme4*^27^; LMM_1_) to test how tempo (log-transformed) varies with phylogenetic distance, using individual age in months as a control factor, and including a random intercept for file to account for variation across recordings. A second model (LMM_2_) included the interaction between phylogenetic distance and behavioral context in which laughter took place (play/tickling). We calculated slopes for each level of the factor “context” with the *emtrends* function (*emmeans* package^28^). A third model (LMM_3_) was fitted to test how tempo varies within context for each species, with laughter tempo (log-transformed) as the response variable, as fixed factors the interaction between species identity and context (play/tickling), individual age as a control factor, and file identity as a random intercept. We subsequently performed pairwise comparisons for each level species x context with the *emmeans* package^28^.

### Tempo variability

We then calculated the coefficient of variation (CV) of laughter tempo for each recording (*N* = 32) and used this value as the response variable. We fitted a fourth model (LMM_4_) with phylogenetic distance and age as fixed factors and individual identity as a random intercept to account for repeated measurements. We extracted p-values for each predictor using the R *summary* function. The limited sample size prevented us from investigating potential differences between the two contexts.

### Testing isochrony in laughter

Finally, to assess the rhythmic structure of laughter, we computed *r*_*k*_ for each pair of successive *t*_*k*_ as the duration of a given *t*_*k*_ relative to the combined duration of that *t*_*k*_ and the following one. By doing so, we obtained 316 *r*_*k*_ values (bonobos 70, chimpanzees 81, gorillas 78, humans 43, and orangutans 44). Following previous works^13,29^, we tested the significance of isochrony peaks (*r*_*k*_ = 0.5) by counting the number of *r*_*k*_ values falling within the on-isochrony range (0.440 < *r*_*k*_  < 0.555) and the off-isochrony ranges (0.400 < *r*_*k*_ < 0.440 and 0.555 < *r*_*k*_ < 0.600), which are symmetrically distributed around the 1:1 ratio. To test whether rhythmic ratios (*r*_*k*_) were more likely to fall within the on-integer ratio range than within the off-integer ratio ranges, we fitted three Generalized Linear Mixed Models (*glmmTMB* package^30^), using a Poisson distribution (count data) and specifying *ziformula* = 1 to account for excess zeros. All models included an offset to weight *r*_*k*_ counts by bin width. The first model (GLMM_1_) contained the *r*_*k*_ count as the response variable, the type of bin in which the observations fell (two levels: on-isochrony or off-isochrony) as a predictor, and the individual identity of the subject, its species, and age (in months) as nested random factors. We obtained *p*-values by applying a post-hoc test (*emmeans*^28^). The other two models aimed at investigating possible differences in rhythmic structure depending on context, with the same syntax as the first one, but investigating two different subsets. The second model (GLMM_2_) aimed at analyzing only the *r*_*k*_ produced in the context of play: this is the only case where the full model did not significantly differ from the null one, indicating that bin identity (on vs off isochrony) does not explain the variability of the response variable. The third model (GLMM_3_) analyzed laughter produced in the context of tickling, and we obtained p-values by applying a post-hoc test (*emmeans*^28^).

### Statistics and reproducibility

All statistical analyses were performed in R^24^. Detailed descriptions of statistical models are provided in the relevant Methods subsections. Sample sizes are reported throughout the manuscript and correspond to independent laughter bouts, interval measurements (*t*_*k*_), rhythmic ratios (*r*_*k*_), or recordings, depending on the analysis. Replicates were defined as independent bouts or recordings, and individual and file identity were included as random effects where appropriate to account for repeated measurements. No data were excluded other than recordings that did not meet the predefined inclusion criterion of containing at least three calls per bout. No statistical methods were used to predetermine sample size.

### Reporting summary

Further information on research design is available in the Nature Portfolio Reporting Summary linked to this article.

## Supplementary information

Transparent Peer Review file

Supplementary Information

Reporting Summary

## Data Availability

All datasets supporting the findings of this study are publicly available at Zenodo: 10.5281/zenodo.19005404 (ref. ^31^).

## Code availability

All analysis scripts used to generate the results and figures in this study are publicly available at Zenodo: 10.5281/zenodo.19005404 (ref. ^31^).

## References

[1] Davila-Ross, M., Allcock, B., Thomas, C. & Bard, K. A. Aping expressions? Chimpanzees produce distinct laugh types when responding to laughter of others. *Emotion* **11**, 1013–1020 (2011). 21355640 10.1037/a0022594

[2] Provine, R. R. *Laughter: A Scientific Investigation*. xi, 258 (Penguin Press, New York, NY, US, 2001).

[3] Davila-Ross, M. D., Owren, M. J. & Zimmermann, E. The evolution of laughter in great apes and humans. *Commun. Integr. Biol.* **3**, 191–194 (2010). 20585520 10.4161/cib.3.2.10944 PMC2889984

[4] Davila-Ross, M. J., Owren, M. & Zimmermann, E. Reconstructing the evolution of laughter in great apes and humans. *Curr. Biol.* **19**, 1106–1111 (2009). 19500987 10.1016/j.cub.2009.05.028

[5] Davila-Ross, M. & Palagi, E. Laughter, play faces and mimicry in animals: evolution and social functions. *Philos. Trans. R. Soc. B Biol. Sci.* **377**, 20210177 (2022). 10.1098/rstb.2021.0177 PMC9489294 36126662

[6] Freeberg, T. M., Dunbar, R. I. M. & Ord, T. J. Social complexity as a proximate and ultimate factor in communicative complexity. *Philos. Trans. R. Soc. Lond. B Biol. Sci.* **367**, 1785–1801 (2012). 22641818 10.1098/rstb.2011.0213 PMC3367695

[7] McComb, K. & Semple, S. Coevolution of vocal communication and sociality in primates. *Biol. Lett.* **1**, 381–385 (2005). 17148212 10.1098/rsbl.2005.0366 PMC1626386

[8] Lameira, A. R., Hardus, M. E., Ravignani, A., Raimondi, T. & Gamba, M. Recursive self-embedded vocal motifs in wild orangutans. *eLife* **12**, RP88348 (2024). 38252123 10.7554/eLife.88348 PMC10945596

[9] De Gregorio, C., Gamba, M. & Lameira, A. R. Third-order self-embedded vocal motifs in wild orangutans, and the selective evolution of recursion. *Ann. N. Y. Acad. Sci.* **1549**, 219–229 (2025). 40376956 10.1111/nyas.15373 PMC12309442

[10] De Gregorio, C. & Lameira, A. R. Twice times two: dual mechanism for double rhythmic meter in orangutans and the evolution of human song. *iScience* **29**, 114273 (2026). 41492465 10.1016/j.isci.2025.114273 PMC12765161

[11] De Gregorio, C. et al. Categorical rhythms in a singing primate. *Curr. Biol.* **31**, R1379–R1380 (2021). 34699799 10.1016/j.cub.2021.09.032

[12] De Gregorio, C. et al. Isochrony as ancestral condition to call and song in a primate. *Ann. N. Y. Acad. Sci.* **1537**, 41–50 (2024). 38925552 10.1111/nyas.15151

[13] De Gregorio, C., Antonini, P., Heymann, E. W. & Gamba, M. Isochrony in titi monkeys duets: social context as a proximate cause of duets’ rhythm and regularity. *Proc. R. Soc. B: Biol. Sci.* **292**, 20242805 (2025). 10.1098/rspb.2024.2805 PMC11836696 39968619

[14] Ma, H. et al. Small apes adjust rhythms to facilitate song coordination. *Curr. Biol.* **34**, 935–945.e3 (2024). 38266649 10.1016/j.cub.2023.12.071

[15] De Gregorio, C. et al. Isochronous singing in 3 crested gibbon species (Nomascus spp.). *Curr. Zool.* **70**, 291–297 (2024). 39035758 10.1093/cz/zoad029 PMC11255994

[16] Pellis, S. M., Pellis, V. C. & Reinhart, C. J. The Evolution of Social Play. in *Formative Experiences: The Interaction of Caregiving, Culture, and Developmental Psychobiology* (eds Worthman, C. M., Cummings, C. A., Schechter, D. S. & Plotsky, P. M.) 404–431 (Cambridge University Press, Cambridge, 2010). 10.1017/CBO9780511711879.037.

[17] Palagi, E. et al. Rough-and-tumble play as a window on animal communication. *Biol. Rev.* **91**, 311–327 (2016). 25619897 10.1111/brv.12172

[18] Bramble, D. M. & Carrier, D. R. Running and breathing in mammals. *Science* **219**, 251–256 (1983). 6849136 10.1126/science.6849136

[19] Kipper, S. & Todt, D. Variation of sound parameters affects the evaluation of human laughter. *Behaviour* **138**, 1161–1178 (2001).

[20] Kipper, S. & Todt, D. The role of rhythm and pitch in the evaluation of human laughter. *J. Nonverbal Behav.* **27**, 255–272 (2003).

[21] Echols, C. H., Crowhurst, M. J. & Childers, J. B. The perception of rhythmic units in speech by infants and adults. *J. Mem. Lang.* **36**, 202–225 (1997).

[22] Gabrielsson, A. (ed) *Action and Perception in Rhythm and Music: Papers given at a Symposium in the Third International Conference on Event Perception and Action*. 237 (Royal Swedish Academy of Music, 1987).

[23] Vettin, J. & Todt, D. Human laughter, social play, and play vocalizations of non-human primates: an evolutionary approach. *Behaviour* **142**, 217–240 (2005).

[24] R Core Team. R. R: A language and environment for statistical computing. R Foundation for Statistical Computing, Vienna, Austria. https://www.R-project.org/ (2021).

[25] Kumar, S., Stecher, G., Suleski, M. & Hedges, S. B. TimeTree: a resource for timelines, timetrees, and divergence times. *Mol. Biol. Evol.* **34**, 1812–1819 (2017). 28387841 10.1093/molbev/msx116

[26] Dobson, A. J. *An Introduction to Generalized Linear Models* (Chapman and Hall/CRC, New York, 2001). 10.1201/9781420057683.

[27] Bates, D., Mächler, M., Bolker, B. & Walker, S. Fitting linear mixed-effects models using lme4. *J. Stat. Softw.* **67**, 1–48 (2015).

[28] Lenth, R. emmeans: Estimated Marginal Means, aka Least-Squares Means_. R package version 1.8. 5. (2023).

[29] Roeske, T. C., Tchernichovski, O., Poeppel, D. & Jacoby, N. Categorical rhythms are shared between songbirds and humans. *Curr. Biol.* **30**, 3544–3555.e6 (2020). 32707062 10.1016/j.cub.2020.06.072 PMC7511425

[30] Brooks, M. E. et al. glmmTMB balances speed and flexibility among packages for zero-inflated generalized linear mixed modeling. *R. J.* **9**, 378–400 (2017).

[31] De Gregorio, C., Davila-Ross, M., & Lameira, A. (2026). Rhythm and timing in laughter reveal that human vocal plasticity falls on a hominid continuum [Data set]. Communications Biology. *Zenodo*. 10.5281/zenodo.19005404. 10.1038/s42003-026-10499-z PMC13304170 42350767

