# Supplementary material for: Rhythm and timing in laughter reveal that human vocal plasticity falls on a hominid continuum
Source: Commun Biol. 2026 Jun 25;9:824. doi: 10.1038/s42003-026-10499-z (PMC13304170; doi:10.1038/s42003-026-10499-z)
Supplement: Supplementary file 2 — Supplementary Information [file 42003_2026_10499_MOESM2_ESM.pdf]

Supplementary Tables – Results of statistical modeling

Model Number	Contrast	Estimate	SE	z.ratio	p value
**GLMM$_1$**	11off vs 11on	-0.391	0.0166	-2.531	0.019
**GLMM$_3$**	11off vs 11on	-0.470	0.2130	-2.205	0.0275

Supplementary Table 1. Results of the post hoc comparisons of the GLMM1 and GLMM3 testing for the effect of r_k bin type (11off, 11on) on the r_k count. GLMM$_1$, Null vs. Full—χ2 = 5.15772, df = 1, p = 0.023; GLMM$_2$ is not reported because Null vs. Full—χ2 = 0.778161, df = 1, p = 0.377; GLMM$_3$, Null vs. Full—χ2 = 4.716765, df = 1, p = 0.030. Model syntax: *glmmTMB(sum ~ bins2 + offset(offset) + (1|id/species/age_months),ziformula = ~1, data=m, family=poisson()).*

LMM$_1$ *lmer(formula = log_dur ~ phylo_distance + age_months+ (1|file), data = b_age_months*

Factor	estimate	SE	df	t value	p value
phylo_distance	0.067987	0.012861	27.586049	5.286	<0.001
age_months	0.010992	0.003627	31.693621	3.031	0.005

Supplementary Table 2. Results of the model testing for the influence of phylogenetic distance on laughter tempo. LMM1: Null vs. Full—χ2 = 27.24386, df = 2, P < 0.001.

a) LMM$_2$ *lmer(formula = log_dur ~ phylo_distance * context + age_months+ (1|file)*

Context	phylo_distance trend	SE	df	Lower. CL	Upper. CL	t.ratio	p value
Play	0.0176	0.0234	41.7	-0.0297	0.0649	0.751	0.457
Tickling	0.0870	0.0152	42.9	0.0564	0.1176	5737	**<0.001**

b) LMM$_3$ *lmer(formula = log_dur ~ species * context + age_months+ (1|file)*

Species	contrast	estimate	SE	df	Lower. CL	Upper. CL	t.ratio	p value
Chimp	Play vs Tickling	-0.0213	0.332	53.4	-0.687	0.644	-0.064	0.949
Gorilla	Play vs Tickling	-0.2299	0.277	56.1	-0.785	0.325	-0.830	0.410
Human	Play vs Tickling	0.964	0.377	48.6	0.207	1.721	2.559	**0.014**
Orangutan	Play vs Tickling	-0.2054	0.284	59.1	-0.774	0.363	-0.723	0.474

c) LMM4 *lmer(formula = cv_tk ~ phylo_distance + age_months+ (1|id), data = cv_file, REML=F)*

Fixed effects	estimate	SE	df	t.value	pvalue
phylo_distance	-2.4336	0.7527	18.1881	-3.233	**0.004**
age_months	-0.1804	0.2067	20.1341	-0.873	0.393

Supplementary Table 3. a) Result of the model testing for the influence of phylogenetic distance and context on laughter tempo in great apes. LMM2: Null vs. Full—χ2 = 34.22303, df = 4, p < 0.001; b) Results of the post hoc comparisons between laughter context in great aspes. LMM3: Null vs. Full—χ2 = 827.7109, df = 9, p< 0.001; c) Result of the model testing for the influence of phylogenetic distance on the coefficient of variation of laughter tempo in great apes. LMM4: Null vs. Full—χ2 = 8.971765, df = 2, p = 0.011.

Species	CV t_k	SD CV t_k	SE CV t_k
Bonobo	52.0	27.3	11.1
Chimp	42.1	18.8	7.68
Gorilla	34.5	15.0	5.67
Human	77.4	20.2	9.04
Orangutan	35.2	14.2	5.01

Supplementary Table 4. Coefficient of variations of laughter tempo (t_k) per species, their standard deviation and standard error

Species	Id	Sex	Age (months)	Location	Country
**Orangutan**	Kajan	male	6 & 19	Zoo Wilhelma	Germany
	Aris	male	24	Zoo Wilhelma	Germany
	Tobby	male	36	Sepilok Rehabilitation center	Malaysia
	Rosa	female	36	Sepilok Rehabilitation center	Malaysia
**Gorilla**	Ndowe	male	10	Zoo Wilhelma	Germany
	Po	male	8	Allwetterzoo Muenster	Germany
**Bonobo**	Yasongo	male	9 & 18	Zoo Wuppertal	Germany
	Congo	male	48	Zoo Frankfurt	Germany
	Mai	male	84	Zoo Wilhelma	Germany
**Chimpanzee**	Joe	male	8	Zoo Hannover	Germany
	Pepe	male	10	Zoo Hannover	Germany
	Mike	male	20	Schwaben Park	Germany
	Male	male	48	Allwetterzoo Muenster	Germany
**Human**	Kid 1	male	36	Private home	Germany
	Kid 2	male	12	Private home	Germany
	Kid 3	male	15	Private home	Germany
	Kid 4	male	11	Private home	Germany

Supplementary Table 5. Information on species, individual identity, sex, age, and recording location of the subjects.

Supplementary Figure

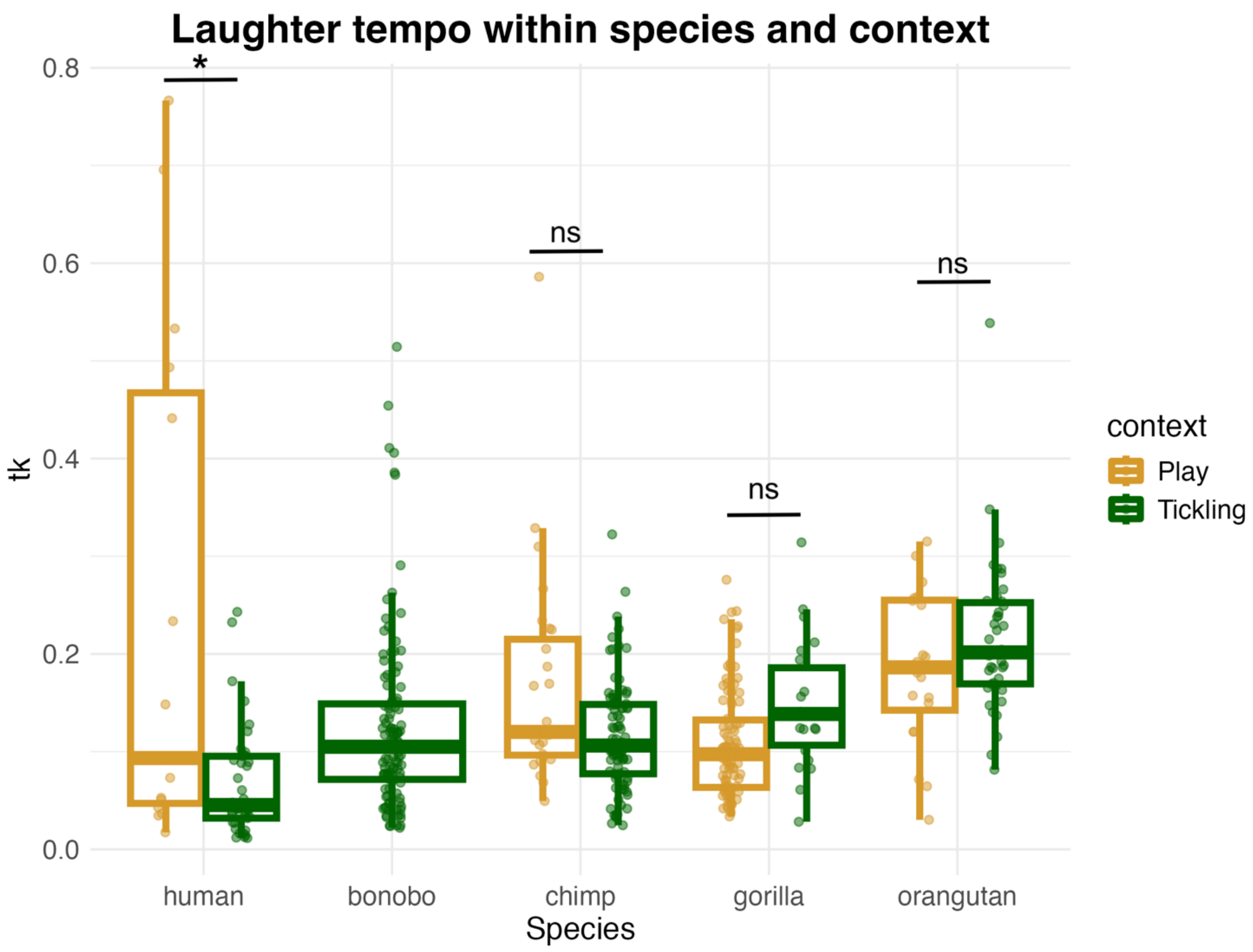

Supplementary Figure S1. Boxplot showing tempo (t_k) of laughter between different context of emission per species. Bonobo recordings occurred in the tickling context only. *Denotes p = 0.014; ns = not significant. Dots represents datapoints. Sample sizes are as follows: n = 4 biologically independent animals for orangutans, n = 2 for gorillas, n = 3 for bonobos, n = 4 for chimpanzees, and n = 4 children. Statistical inference is based on mixed-effects models; see Method.
