# Supplementary material for: Rhythm and timing in laughter reveal that human vocal plasticity falls on a hominid continuum
Source: Commun Biol. 2026 Jun 25;9:824. doi: 10.1038/s42003-026-10499-z (PMC13304170; doi:10.1038/s42003-026-10499-z)
Supplement: Supplementary file 3 — Reporting Summary [file 42003_2026_10499_MOESM3_ESM.pdf]

nature portfolio

Corresponding author(s): Chiara De Gregorio

Last updated by author(s): 05/05/2026

Reporting Summary

Nature Portfolio wishes to improve the reproducibility of the work that we publish. This form provides structure for consistency and transparency in reporting. For further information on Nature Portfolio policies, see our Editorial Policies and the Editorial Policy Checklist.

Statistics

For all statistical analyses, confirm that the following items are present in the figure legend, table legend, main text, or Methods section.

n/a	Confirmed	
☐	☒	The exact sample size (*n*) for each experimental group/condition, given as a discrete number and unit of measurement
☒	☐	A statement on whether measurements were taken from distinct samples or whether the same sample was measured repeatedly
☐	☒	The statistical test(s) used AND whether they are one- or two-sided<br>*Only common tests should be described solely by name; describe more complex techniques in the Methods section.*
☐	☒	A description of all covariates tested
☐	☒	A description of any assumptions or corrections, such as tests of normality and adjustment for multiple comparisons
☐	☒	A full description of the statistical parameters including central tendency (e.g. means) or other basic estimates (e.g. regression coefficient) AND variation (e.g. standard deviation) or associated estimates of uncertainty (e.g. confidence intervals)
☐	☒	For null hypothesis testing, the test statistic (e.g. *F*, *t*, *r*) with confidence intervals, effect sizes, degrees of freedom and *P* value noted<br>*Give P values as exact values whenever suitable.*
☒	☐	For Bayesian analysis, information on the choice of priors and Markov chain Monte Carlo settings
☒	☐	For hierarchical and complex designs, identification of the appropriate level for tests and full reporting of outcomes
☒	☐	Estimates of effect sizes (e.g. Cohen's *d*, Pearson's *r*), indicating how they were calculated

Our web collection on statistics for biologists contains articles on many of the points above.

Software and code

Policy information about availability of computer code

Data collection	na
Data analysis	R Software

For manuscripts utilizing custom algorithms or software that are central to the research but not yet described in published literature, software must be made available to editors and reviewers. We strongly encourage code deposition in a community repository (e.g. GitHub). See the Nature Portfolio guidelines for submitting code & software for further information.

Data

Policy information about availability of data

All manuscripts must include a data availability statement. This statement should provide the following information, where applicable:

- Accession codes, unique identifiers, or web links for publicly available datasets
- A description of any restrictions on data availability
- For clinical datasets or third party data, please ensure that the statement adheres to our policy

Code and dataset associated with this MS was uploaded during the submission process

Research involving human participants, their data, or biological material

Policy information about studies with human participants or human data. See also policy information about sex, gender (identity/presentation), and sexual orientation and race, ethnicity and racism.

Reporting on sex and gender	na
Reporting on race, ethnicity, or other socially relevant groupings	na
Population characteristics	na
Recruitment	na
Ethics oversight	na

Note that full information on the approval of the study protocol must also be provided in the manuscript.

Field-specific reporting

Please select the one below that is the best fit for your research. If you are not sure, read the appropriate sections before making your selection.

☐ Life sciences ☐ Behavioural & social sciences ☒ Ecological, evolutionary & environmental sciences

For a reference copy of the document with all sections, see nature.com/documents/nr-reporting-summary-flat.pdf

Ecological, evolutionary & environmental sciences study design

All studies must disclose on these points even when the disclosure is negative.

Study description	We compared temporal features of laughter across four non human primaes and humans, using rhythm and its variability as a proxy of vocal flexibility.
Research sample	4 orangutans- Pongo pygmaeus (3 males and 1 female); 2 gorillas - Gorilla gorilla (males); 3 bonobos - Pan paniscus (males); 4 chimpanzees (Pan troglodytes); 4 humans - Homo sapiens (males). Details in table SM5
Sampling strategy	Data were collected ad libitum during spontaneous play interaction and tickling induced interaction.
Data collection	Audio recordings were collected from four species of non-human primates (four orangutans, two gorillas, three bonobos, and four chimpanzees) at seven different institutions. Additionally, we collected recordings of laughter from kids in their private homes. Details of ID, sex, age, and location of recording can be found in Table SM5. All subjects were acoustically recorded in their home environments during controlled, playful interactions with familiar and unfamiliar humans, who elicited both play and tickle-induced vocalizations. Most of the tickling recordings from non-human apes were previously included in Davila-Ross and colleagues. This procedure has been reliably employed to investigate spontaneous vocal behavior across multiple species15. Recordings were made using a Sony WM-D6C with a Sennheiser ME 60 microphone and a Nagra IV-SJ with a Sennheiser MKH 816 microphone.
Timing and spatial scale	Part of the data used in this work were originally collected by Davila-Ross and colleagues (2009), between 2004-2006.
Data exclusions	na
Reproducibility	We provided detailed methodology, the syntax of all models, the dataset and code used for the analyses.
Randomization	na
Blinding	na

Did the study involve field work? ☒ Yes ☐ No

Field work, collection and transport

Field conditions	Data were collected in 9 different zoos across Germany and Asia and private homes (for humans).
Location	Zoo Wilhelma, Allwetterzoo Muenster, Zoo Wuppertal , Zoo Frankfurt, Zoo HannoverSchwaben Park (Germany), Sepilok Rehabilitation center (Malaysia).

Access & import/export	na
Disturbance	na

Reporting for specific materials, systems and methods

We require information from authors about some types of materials, experimental systems and methods used in many studies. Here, indicate whether each material, system or method listed is relevant to your study. If you are not sure if a list item applies to your research, read the appropriate section before selecting a response.

Materials & experimental systems

n/a	Involved in the study
☒	☐ Antibodies
☒	☐ Eukaryotic cell lines
☒	☐ Palaeontology and archaeology
☐	☒ Animals and other organisms
☒	☐ Clinical data
☒	☐ Dual use research of concern
☒	☐ Plants

Methods

n/a	Involved in the study
☒	☐ ChIP-seq
☒	☐ Flow cytometry
☒	☐ MRI-based neuroimaging

Animals and other research organisms

Policy information about studies involving animals; ARRIVE guidelines recommended for reporting animal research, and Sex and Gender in Research

Laboratory animals	Zoo housed great apes
Wild animals	na
Reporting on sex	Due to the sample size, account for possible sex differences was not feasible.
Field-collected samples	na
Ethics oversight	Data were collected between 2004-2006. At the time ethical approval was not required.

Note that full information on the approval of the study protocol must also be provided in the manuscript.

Plants

Seed stocks	na
Novel plant genotypes	na
Authentication	na
