# Supplementary material for: Rhythm and timing in laughter reveal that human vocal plasticity falls on a hominid continuum
Source: Commun Biol. 2026 Jun 25;9:824. doi: 10.1038/s42003-026-10499-z (PMC13304170; doi:10.1038/s42003-026-10499-z)
Supplement: Supplementary file 1 — Transparent Peer Review file [file 42003_2026_10499_MOESM1_ESM.pdf]

nature portfolio

Peer Review File

Rhythm and timing in laughter reveal that human vocal plasticity falls on a hominid continuum

Corresponding Author: Dr Chiara De Gregorio

This manuscript has been previously submitted at another journal. This document only contains information relating to versions considered at Communications Biology.

This file contains all reviewer reports in order by version, followed by all author rebuttals in order by version.

Version 0:

Reviewer comments:

Reviewer #1

(Remarks to the Author)
This is a novel and important study on the variability of laughter across species. I find this research particularly compelling because it compares multiple species to enhance our understanding of evolutionary trends in communication, an aspect that many current studies neglect. While I think this study is important and that the content is overall good, additional clarifying information is required in the main text and methods sections before publication.

Brief, unreferenced abstract: I think you should elaborate on how laughter serves as a valuable proxy for understanding the evolution of vocal control. I know you mention this in the actual text, but for clarity, it should be laid out in the brief abstract for your readers.

MAIN TEXT: I will begin by discussing general points before providing line-by-line comments. While the introduction has good content, it lacks sufficient references. You make several broad claims about significant evolutionary patterns and the communicative abilities of animals, but you need additional evidence to support them. For instance, the first paragraph contains only two references, yet multiple sentences should have citations (see lines 20-23, 26-27, 27-28, etc.). While I understand this may be a brief communication (limited to 2,000-5,000 words), it is still essential to reference this information. Utilizing review articles could be a good compromise in this case. Additionally, your methodological information is too vague, and your results are difficult to interpret because you don't clearly identify the metrics associated with your statistical models. I do like Figure 1, though!

Specific feedback:
1. Line 20-21: This is likely due to their unique socio-ecologies, correct? Might be good to briefly mention here. Additionally, at the end of this sentence, please include evidence (i.e., citations) regarding laughter in great apes.
2. Line 23-24: Not sure what you are trying to say here… You could probably just omit altogether.
3. Line 28: Elaborate more on what you mean by "reflecting major trends and transformations".
4. Line 33: Why social play and tickling contexts only? I recognize that the answer to this is quite intuitive, but it's important to discuss why these two have been chosen. This also provides an opportunity to reference the relevant research literature.
5. Line 35: Ok, but why should we expect to see variability? This is why I think a discussion of how socio-ecological conditions shape communicative repertoires is necessary.
6. Lines 39-41: I looked at the electronic supplement, and it appears that most of your samples are coming from a captive environment (Zoo Wilhelma). It's good to mention this, as this could be an important study limitation to discuss later in the methods section.
7. Lines 43-46: You mentioned "tk" as a way to operationalize laughter tempo. While I understand that two other variables contribute to this statistic, I believe you should provide more clarifying details on how this metric actually works in this section. I reviewed the methods section and found it lacking clarity on "tk" as well.
8. Line 50: After reading this paragraph and looking at the electronic supplement, it appears that you are running multiple sets of GLMMs, some that group all apes together and others that account for differences in call production based on ape type. It might be good to mention in the text so your readers can follow along.
9. Line 52: Should humans be examined separately from non-human great apes if you are making this sort of claim? The

model seems to include all of them together, differentiating only by a random factor. Perhaps you can rephrase this sentence to better reflect the models you were using.
10. Lines 56-58: Put a citation here.
11. Line 59: Needs a citation.
12. Line 69: What do you mean by positive here? You are contrasting this term with “rigid, stereotyped timing”, so perhaps you mean to say flexibility here?

FIGURE 1: This is great!!!

METHODS: A great deal of clarifying information is needed here. While you reference previous studies that inform the current study, it's essential to describe their data and methodologies in detail here as well. This way, your readers won't have to search for those publications to understand your study. They may not have access to those studies, after all. See below for my line-by-line comments…

Specific feedback:
1. Line 95: Report sample sizes for humans, just as you have done for the other great apes. Also, these sample sizes seem really small for addressing such big-picture evolutionary questions. My guess is that this is because getting access to such young non-human great apes is hard, as most captive populations are comprised mostly of adults. It might be helpful to mention this so your readers appreciate the work you have done.
2. Line 96-97: Why also unfamiliar individuals? As I am guessing this wasn’t the case for your non-human great ape samples. Seems to me like you would prefer to gather recordings from spontaneous bouts of laughter during play and tickling with familiar individuals. This could explain the greater variability in your results with humans. Do you think that including data on unfamiliar interactions in non-human great apes would yield similar results?
3. Line 99: You are referring to the data points in the other studies, correct? Or are you referring to coding protocols? Be clearer here.
4. Line 101: How do you operationalize play and tickling? Relates to my comment for line 99.
5. Line 101: What sampling method did you use? Did you continuously record videos? Or only recorded instances of laughter-based interactions?
6. Lines 113-114: The sample sizes seem really small for humans, which is surprising. Is this because of differences in the number of recording hour opportunities?
7. Line 120-121: How was this done?
8. Line 146: You need to clarify how “tk” was calculated before you can discuss “rk”. See comment above for the main text.

Reviewer #2

(Remarks to the Author)
“Rhythmic evolution of laughter shows human vocal plasticity falls on a hominid continuum”
De Gregorio et al.

This study investigates the evolution of laughter's temporal structure by analyzing acoustic recordings from five hominid taxa: orangutans, gorillas, bonobos, chimpanzees, and humans. Using inter-onset intervals and rhythmic ratios, the authors report that laughter is isochronous across all great apes, a trait conserved from the last common ancestor. The study claims that while the fundamental rhythm (i.e., isochrony) is conserved, the tempo has accelerated and the variability of the rhythm has increased along the phylogenetic line toward humans. The authors conclude that these changes represent an "evolution of vocal control" and "rhythmic plasticity" that served as a precursor to speech.
The manuscript presents an interesting comparative dataset, particularly in its attempt to quantify the temporal characteristics of laughter across hominids.
However, there is a critical semantic and theoretical disconnect and between the results and the manuscript's title/framing. Since "isochrony" is conserved, the term "Rhythmic Evolution" in the title is misleading. What has evolved is not the rhythm itself, but the flexibility to modulate it. Furthermore, the mechanism driving this plasticity is likely rooted in respiratory control rather than purely vocal motor control.
Therefore, I think the manuscript is suitable for publication only if the following revisions are made:
1. Title & Framing: Change the title to reflect that rhythm is conserved while plasticity/flexibility evolved.
2. Mechanism: Explicitly discuss that the increased plasticity in humans is mechanically enabled by the shift from alternating (panting) to sustained egressive airflow (e.g., Davila Ross et al., 2009).
3. Limitations: Add a clear caveat regarding the small sample sizes (N=2–3) for non-human apes, noting that estimates of species-level variability (CV) are preliminary.
If the authors are unable to reframe the narrative from "Rhythmic Evolution" to "Evolution of respiratory control," or cannot address the mechanical definition of control, I recommend declining the submission to allow for transfer to a more specialized journal.

The following are comments on the major points related to the above:

1. The Title and "Rhythmic Evolution"
The title "Rhythmic evolution... shows human vocal plasticity" is misleading because the study actually finds that the rhythmic category (isochrony) is conserved (did not evolve) and what changed was tempo and coefficient of variation (CV). The title implies a structural change in rhythm that the data does not support. A more accurate title should reflect the difference of tempo and variation across hominids.

2. Misinterpretation of "Vocal Control" vs. "Respiratory Constraints"
The authors attribute the increase in rhythmic variability to "enhanced vocal control". However, the data and the previous literatures (e.g., Davila Ross et al., 2009; 2010) strongly suggest that this is actually a result of respiratory control.
For example, Davila Ross et al., (2009) reports that great ape laughter is predominantly "alternating egressive-ingressive" (panting). This means the "rhythm" of ape laughter is physically bound to the respiratory cycle. In contrast, humans produce "exclusively egressive" laughter, segmenting a single exhalation into multiple calls. Therefore, the observed "isochrony" in apes likely reflects the default output of the respiratory Central Pattern Generator (CPG). Actually, the authors themselves acknowledge in the manuscript that play disrupts the rhythm because it involves "compressing of the thorax" and disrupts the "respiratory cycle".
So, the "evolution" observed is not necessarily the acquisition of vocal rhythmic ability, but rather, it is reasonable to think that the decoupling of vocalization from the strict 1:1 respiratory cycle. Humans show high variability because they can cognitively override the respiratory CPG, whereas apes are physiologically constrained by the panting mechanic. I think that the manuscript should reframe the discussion to address Respiratory Control/Flexibility rather than purely "Vocal Control."

3. Inadequacy of Sample Size for Evolutionary Claims
The sample sizes are insufficient to draw robust conclusions about species-specific evolutionary trends, particularly regarding "variability" and "plasticity," which are highly subject to individual differences. While the authors use GLMMs to account for individual identity , the statistical power relies heavily on the number of calls rather than the number of independent biological units (individuals). The claim that variability decreases with phylogenetic distance is weak when the datapoints representing "distance" are based on so few individuals.

4. Methodological Concerns: Measuring Onsets in "Noisy" Signals
The validity of the rhythm analysis depends entirely on the precise measurement of the call onset. Davila Ross et al. (2009) describes ape laughter as "overwhelmingly irregular and noisy". Unlike the clear, voiced harmonics of human laughter, ape laughter is often a breathy pant.
Indeed, the Davila Ross et al. (2009) (particularly concerning orangutan tickling) also includes spectrograms where the onset is difficult to identify. Therefore, the author should describe the method to demonstrate that the onset of the analyzed vocalization can be accurately detected.

Reviewer #3

(Remarks to the Author)
De Gregorio and colleagues have prepared a very interesting short manuscript on their analysis of the rhythmic properties of laughter across several great ape species. I found the paper to be well written and of sufficient relevance and potential impact for the journal's general readership.

I have just two small comments about the methods on which I would appreciate further explanation/consideration by the authors:
- The use of infant humans is a good idea, not only in matching the age range of the existing sample but also because older human children exhibit forms of volitional laughter reflective of emergent social strategies (e.g. echoing or mimicking adults: the example of when small children laugh in chorus with adults without knowing the reason for the laughter). In the sample reported in the paper, I however see that one individual is 36 months old and was wondering if their behaviours may be more reflective of these kinds of social developments than the substantially younger infants (11, 12, and 15mo). Can the authors justify the inclusion of this child as developmentally equivalent to the other 3 individuals?
- I noticed that the individual identity was modelled as a random effect for some, but not all of the analyses. Can the authors please explain their decision-making around this? In the above case, for example, the 36 month old could be showing more sophisticated/complex behaviours compared to the younger individuals that could play a role in driving inter-species effects.

In Figure 1, I would recommend making more distinct the on- vs off-ratio ranges (e.g. using different colours) and giving some explanation of those in the figure legend so that they can be understood without reading the Methods.

Version 1:

Reviewer comments:

Reviewer #1

(Remarks to the Author)
Thank you for addressing all my comments & concerns! I look forward to reading and citing the final version of this article in my own work. :)

Comments for rebuttal to Reviewer 2:

I have reviewed the responses to reviewer #2. In my professional opinion, the changes made to most of the points are reasonable, and the authors have even changed the title. The methodological concerns raised by reviewer #2 are valid, particularly regarding sample sizes. This challenge is common in my field, but the authors appropriately employ the necessary frequentist statistical methods to address it.

The only feedback that the authors did not adequately address in their revisions & responses was point #4, which pertains to the 'noisiness' of the signals. I believe the authors should acknowledge the presence of 'noisiness' in their manuscript and explain how each protocol helps minimize the confounding factors present in these recordings.

Reviewer #3

(Remarks to the Author)
This is overall a very nice paper and I'm not going to contest anything further. However, I would have been more satisfied if the authors could have reported - if even only for the letter - an analysis that excluded the 36-month-old human participant. While I will admit that my initial query had focused on the older child's social development and its possible influence on laughter behaviours - and I'm satisfied that the authors checked the study samples were play-induced behaviours - I'm not convinced by the argument in the rebuttal that the older child (who at 36mo would likely be verbal) holds comparable relevant vocal and motor abilities to 11-15mo children (who could be preverbal or only uttering first words).

Open Access This Peer Review File is licensed under a Creative Commons Attribution 4.0 International License, which permits use, sharing, adaptation, distribution and reproduction in any medium or format, as long as you give appropriate credit to the original author(s) and the source, provide a link to the Creative Commons license, and indicate if changes were made.
In cases where reviewers are anonymous, credit should be given to 'Anonymous Referee' and the source.
The images or other third party material in this Peer Review File are included in the article's Creative Commons license, unless indicated otherwise in a credit line to the material. If material is not included in the article's Creative Commons license and your intended use is not permitted by statutory regulation or exceeds the permitted use, you will need to obtain permission directly from the copyright holder.
To view a copy of this license, visit https://creativecommons.org/licenses/by/4.0/

Reviewers' comments:

Reviewer #1 (Remarks to the Author):

This is a novel and important study on the variability of laughter across species. I find this research particularly compelling because it compares multiple species to enhance our understanding of evolutionary trends in communication, an aspect that many current studies neglect. While I think this study is important and that the content is overall good, additional clarifying information is required in the main text and methods sections before publication.

We thank the reviewer for their positive and encouraging comments. We greatly appreciate the recognition of the novelty and importance of our study, and we have addressed the suggested clarifications in the main text and methods sections as indicated. Your feedback has been very helpful in improving the clarity and presentation of our work.

Brief, unreferenced abstract: I think you should elaborate on how laughter serves as a valuable proxy for understanding the evolution of vocal control. I know you mention this in the actual text, but for clarity, it should be laid out in the brief abstract for your readers.

Thank you for this comment. We agree that the role of laughter as a proxy for the evolution of vocal control could be made more explicit in the brief abstract. We have therefore slightly expanded the abstract to clarify why laughter is particularly informative in this respect, highlighting its conservation across great apes. It now reads (LL 9-16) "Laughter is an important, universal form of human non-linguistic vocal expression and, being shared by all extant great apes, offers a valuable proxy for tracing the evolution of vocal control that ultimately enabled language. Yet surprisingly little is known about the evolution of its defining feature, rhythm. Comparative analyses of laughter across all extant great apes (orangutans, gorillas, bonobos, chimpanzees, humans) show that the laughter of the last common ancestor was already isochronic, becoming faster, more variable, and increasingly context-sensitive over hominid evolution. The evolution of laughter's rhythm reveals a progressive increase in vocal rhythmic plasticity, with humans following the overall trajectory toward enhanced vocal control."

MAIN TEXT: I will begin by discussing general points before providing line-by-line comments. While the introduction has good content, it lacks sufficient references. You make several broad claims about significant evolutionary patterns and the communicative

abilities of animals, but you need additional evidence to support them. For instance, the first paragraph contains only two references, yet multiple sentences should have citations (see lines 20-23, 26-27, 27-28, etc.). While I understand this may be a brief communication (limited to 2,000-5,000 words), it is still essential to reference this information. Utilizing review articles could be a good compromise in this case. Additionally, your methodological information is too vague, and your results are difficult to interpret because you don't clearly identify the metrics associated with your statistical models. I do like Figure 1, though!

We thank the reviewer for these general comments and suggestions. We have revised the manuscript accordingly, adding additional references to support the claims in the Introduction and providing more detailed methodological information. We have also clarified the metrics associated with our statistical models in the Results section. We hope these changes have improved the clarity and completeness of the manuscript.

Specific feedback:
1. Line 20-21: This is likely due to their unique socio-ecologies, correct? Might be good to briefly mention here. Additionally, at the end of this sentence, please include evidence (i.e., citations) regarding laughter in great apes.

Thank you for your suggestion. We agree that mentioning the differences in socio-ecology between species would be important. We rephrased as (LL 20): "While all major branches of the Hominid family have evolved distinct call repertoires shaped by their species-specific socio-ecologies, one vocalization has been conserved across all species and age–sex classes: laughter. In great apes and humans, laughter typically occurs in playful social interactions and is thought to facilitate social bonding and coordination during potentially ambiguous interactions such as rough-and-tumble play (Davila-Ross et al., 2010; Davila-Ross et al., 2011, Provine, 2001)."

We have added the following citations:

- Davila-Ross M, Owren MJ, Zimmermann E. The evolution of laughter in great apes and humans. Commun Integr Biol. 2010 Mar;3(2):191-4. doi: 10.4161/cib.3.2.10944. PMID: 20585520; PMCID: PMC2889984.
- Provine, R. R. (2001). Laughter: A scientific investigation. Penguin.
- Davila-Ross M, Allcock B, Thomas C, Bard KA. Aping expressions? Chimpanzees produce distinct laugh types when responding to laughter of others. Emotion. 2011 Oct;11(5):1013-20. doi: 10.1037/a0022594. PMID: 21355640.

2. Line 23-24: Not sure what you are trying to say here… You could probably just omit altogether.

We deleted this sentence. Thank you.

3. Line 28: Elaborate more on what you mean by “reflecting major trends and transformations”.

Thank you for your comment. We have rephrased this section as (Line 28): “Because laughter in great apes and humans is inherently repetitive and cyclic – “ha-ha-ha-ha” –, variation in its temporal structure and organization may provide a window into evolutionary changes in respiratory–vocal coordination and motor timing across hominids. In particular, differences in the timing, regularity, and flexibility of laughter sequences could reflect broader evolutionary shifts in vocal control and rhythmic flexibility that distinguish genera within the Hominidae.”

4. Line 33: Why social play and tickling contexts only? I recognize that the answer to this is quite intuitive, but it’s important to discuss why these two have been chosen. This also provides an opportunity to reference the relevant research literature.

Thank you for this suggestion. In great apes, laughter is primarily observed in playful interactions, particularly during rough-and-tumble social play and tickling, which are the two contexts in which this vocalization is most reliably elicited and studied. We have now clarified this point in the manuscript and explicitly stated that our analysis focuses on these two principal contexts in which laughter occurs in great apes. We have rephrased as (Line 33): “Earlier studies reported interspecific differences in laughter tempo limited to the tickling context and to a subset of great ape species[3]. In great apes, laughter is most reliably observed during playful interactions, particularly rough-and-tumble social play and tickling (Davila-Ross et al., 2009; Davila-Ross & Palagi, 2022). Building on these observations, here we present the first comparative analysis of laughter rhythm across all extant great apes...”

Added citations:
-Davila-Ross, M., Owren, M. J., & Zimmermann, E. (2009).
Reconstructing the evolution of laughter in great apes and humans. Current Biology.
- Davila-Ross M, Palagi E.; Laughter, play faces and mimicry in animals: evolution and social functions. Philos Trans R Soc Lond B Biol Sci 7 November 2022; 377 (1863): 20210177. https://doi.org/10.1098/rstb.2021.0177

5. Line 35: Ok, but why should we expect to see variability? This is why I think a discussion of how socio-ecological conditions shape communicative repertoires is necessary.

Thank you for this comment. We have clarified that we expect variation in laughter tempo because communicative repertoires are shaped by species-specific socio-ecological conditions, including group size, social structure, and interaction patterns. We now explicitly state in the manuscript that these socio-ecological differences may give rise to variability in the temporal organization of vocalizations across species and contexts (Line 38): "Because communicative repertoires are shaped by species-specific socio-ecological conditions, including group size, social structure, and patterns of interaction (Freeberg et al 2012; McCombe and Semple 2005), variation in the temporal organization of vocalizations may emerge across species and social contexts. By comparing these different types of laughter, we reveal how tempo, its variability, and its regularity have been shaped across species, social contexts, and along the phylogenetic tree."

Added citations:

- Freeberg TM, Dunbar RI, Ord TJ. Social complexity as a proximate and ultimate factor in communicative complexity. Philos Trans R Soc Lond B Biol Sci. 2012 Jul 5;367(1597):1785-801. doi: 10.1098/rstb.2011.0213. PMID: 22641818; PMCID: PMC3367695.
- Karen McComb, Stuart Semple; Coevolution of vocal communication and sociality in primates. *Biol Lett* 22 December 2005; 1 (4): 381–385. https://doi.org/10.1098/rsbl.2005.0366

6. Lines 39-41: I looked at the electronic supplement, and it appears that most of your samples are coming from a captive environment (Zoo Wilhelma). It's good to mention this, as this could be an important study limitation to discuss later in the methods section.

Thank you for your suggestion We rephrased as (line 41): "We recorded laughter bursts from all five extant great ape taxa: four orangutans *(Pongo pygmaeus*), two gorillas (*Gorilla gorilla*), three bonobos (*Pan paniscus*), four chimpanzees (*Pan troglodytes*), and four humans (*Homo sapiens*), aged from six months to seven years old, most of whom were observed in ex situ settings."

7. Lines 43-46: You mentioned "tk" as a way to operationalize laughter tempo. While I understand that two other variables contribute to this statistic, I believe you should provide more clarifying details on how this metric actually works in this section. I reviewed the methods section and found it lacking clarity on "tk" as well.

Thank you for this comment. We agree that the description of *tk* required further clarification. We have therefore rephrased this section to explicitly define *tk* as the interval

from one sound onset to the onset of the following sound, used here as a proxy for laughter tempo (line 51): "t_k, measuring the interval from one sound onset to that of the following one - a proxy for laughter tempo."

8. Line 50: After reading this paragraph and looking at the electronic supplement, it appears that you are running multiple sets of GLMMs, some that group all apes together and others that account for differences in call production based on ape type. It might be good to mention in the text so your readers can follow along.

We thank the reviewer for this helpful suggestion. We have now clarified this point in the Results section. In the preceding paragraph, we specify that *all taxa were included in the same model, with species as a random factor, to evaluate interspecific differences*. We then clarify the subsequent analyses by specifying that species are first modeled along their phylogenetic distance and that the pattern is further examined by context and by species. The revised text now reads (line 72): "In agreement with earlier work, analyses also revealed that laughter rhythm accelerated along hominid evolution (LMM1, $p < 0.001$, Fig. 1B, Tab. SM2), with species modeled along their phylogenetic distance. Notably, this trend was better captured by tickling laughter, than play laughter (LMM2, Play - $p = 0.476$; Tickling - $p < 0.001$; Tab. SM3, Fig. 1C). Indeed, when breaking down laughter tempo by species, only humans modulated laughter tempo according to context (LMM3, Play vs Tickling; $p < 0.014$; Tab. SM3, Fig. SM1), producing faster laughter during tickling than during play. Context-sensitive modulation was absent in great apes."

We hope that this clarification makes the analytical structure easier to follow for readers.

9. Line 52: Should humans be examined separately from non-human great apes if you are making this sort of claim? The model seems to include all of them together, differentiating only by a random factor. Perhaps you can rephrase this sentence to better reflect the models you were using.

Thank you for this suggestion. The model was intentionally designed to include humans and non-human great apes together in order to directly compare their laughter characteristics. However, we agree that the original phrasing might have been unclear, and we have reworded the sentence to better reflect the structure of the model.

We rephrased it as (line 58): "Analyses showed that great apes' laughter is isochronous, that is, involving regular timing between vocal bursts (GLMM1, $p = 0.019$, Tab. SM1). All taxa were included in the same model, with species as a random factor, to evaluate

interspecific differences. This shows that great apes have been laughing in a recognizable way to modern humans for at least 15 million years, extending previous evidence of isochrony in orangutan vocalizations[4],[5] to other great apes."

10. Lines 56-58: Put a citation here.

Thank you for this comment. We have added:
-Pellis, S. M., Pellis, V. C., & Reinhart, C. J. (2010). The evolution of social play. *Formative experiences: The interaction of caregiving, culture, and developmental psychobiology*, 404-431.
-Palagi, E., Burghardt, G. M., Smuts, B., Cordoni, G., Dall'Olio, S., Fouts, H. N., ... & Pellis, S. M. (2016). Rough-and-tumble play as a window on animal communication. *Biological Reviews*, *91*(2), 311-327.

11. Line 59: Needs a citation.

Thank you for this comment. Here, the idea is that the disruption of the respiratory cycle during social play is largely a logical consequence of the dynamic and variable nature of physical interactions. While this has not been directly measured in all great ape play contexts, it is well supported by studies showing that movement and physical exertion modulate respiratory regularity in mammals (e.g., Bramble & Carrier, 1983; Hoffmann et al., 2012). We have added the Bramble & Carrier, 1983 reference in the manuscript.

12. Line 69: What do you mean by positive here? You are contrasting this term with "rigid, stereotyped timing", so perhaps you mean to say flexibility here?

Thank you for this comment. By "positive," we mean that laughter with variable timing is perceived as more socially and emotionally engaging, in contrast to rigid or stereotyped laughter, which is perceived less positively. We have clarified this point in the manuscript to make the distinction between perceived emotional quality and stereotypy explicit. We rephrased as (line 81): "In human laughter, variable timing is perceived as more socially and emotionally positive as opposed to rigid, stereotyped timing…"

FIGURE 1: This is great!!!
Thank you so much!

METHODS: A great deal of clarifying information is needed here. While you reference previous studies that inform the current study, it's essential to describe their data and methodologies in detail here as well. This way, your readers won't have to search for

those publications to understand your study. They may not have access to those studies, after all. See below for my line-by-line comments…

We thank the reviewer for this comment. We have now added detailed information to the Methods section regarding the data and methodologies used in the current study, so that readers can fully understand our approach without needing to consult previous publications. We hope that these revisions have improved clarity and accessibility.

Specific feedback:

1. Line 95: Report sample sizes for humans, just as you have done for the other great apes. Also, these sample sizes seem really small for addressing such big-picture evolutionary questions. My guess is that this is because getting access to such young non-human great apes is hard, as most captive populations are comprised mostly of adults. It might be helpful to mention this so your readers appreciate the work you have done.

Thank you for pointing this out. We have now specified the sample sizes for humans, as we did for the other great apes. We also appreciate your observation regarding the small sample sizes for some species; we have added a sentence clarifying that access to young non-human great apes is limited in most captive populations, which highlights the challenges and effort involved in collecting these data (line 107): "Young non-human great apes are relatively rare in captive populations, which limits sample sizes for this age class."

2. Line 96-97: Why also unfamiliar individuals? As I am guessing this wasn't the case for your non-human great ape samples. Seems to me like you would prefer to gather recordings from spontaneous bouts of laughter during play and tickling with familiar individuals. This could explain the greater variability in your results with humans. Do you think that including data on unfamiliar interactions in non-human great apes would yield similar results?

Thank you for pointing this out. Apologies for the lack of clarity in this sentence. In all cases, the individuals interacting with the subjects were familiar humans. The unfamiliar individual was only the recordist, who was present to record the sessions but did not interact with the subjects. We have now deleted the reference to unfamiliar humans in the manuscript to avoid confusion.

3. Line 99: You are referring to the data points in the other studies, correct? Or are you referring to coding protocols? Be clearer here.

Thank you for this comment. We are indeed referring to the actual data points collected in the previous studies by Davila-Ross and colleagues, not just the coding protocols. We have clarified this point in the manuscript to avoid any ambiguity. It now reads (line 112): “Most of the tickling recordings from non-human apes were previously collected and reported in Davila-Ross and colleagues, and we re-analyzed these original data points for the current study.”

4. Line 101: How do you operationalize play and tickling? Relates to my comment for line 99.

Thank you for your comment. Tickling was part of a playful interaction with a human, including the use of objects, showing self-handicapping and exaggerated movements sometimes. The kind of playful interaction that took place much depended on how the familiar human typically interacted in a playful way with the subject in the past in order to induce laughter. There were no specific instructions provided other than to induce laughter in a playful way.

5. Line 101: What sampling method did you use? Did you continuously record videos? Or only recorded instances of laughter-based interactions?

We thank the reviewer for this question. All individuals were audio-recorded in their home facilities (or homes) during playful interactions in which familiar humans were instructed to elicit tickle-induced vocalizations. For social play, recordings were made ad hoc specifically to capture these interactions. This method targets naturally occurring laughter bouts in a controlled social context and has been successfully applied across multiple primate species. Continuous video recording was not used; instead, we focused on capturing instances of laughter-based interactions as they occurred.

6. Lines 113-114: The sample sizes seem really small for humans, which is surprising. Is this because of differences in the number of recording hour opportunities?

We thank the reviewer for this comment. The apparently small sample size for humans arises from the requirements of our rhythmic analyses: to calculate *tk*, only bouts containing at least two calls can be considered, and to calculate *rk* (which requires two *tk* values), only bouts containing at least three calls are used. These laughter bouts may be shorter in terms of the number of calls, which could explain the lower number of usable datapoints, despite sufficient overall recording time.

7. Line 120-121: How was this done?

Thank you for this comment. The intervals between calls were calculated in R. For each bout, calls were first ordered by their starting time, and then the duration between the starting point of a call and that of the following call within the same bout was computed. The text now reads (line 130): “We used the software R[16] to calculate the duration of the intervals between the starting point…”

8. Line 146: You need to clarify how “tk” was calculated before you can discuss “rk”. See comment above for the main text.

Thank you for this comment. We agree that clarification was needed, and we have now added a clear explanation of how “tk” was calculated before discussing “rk” in the main text.

Reviewer #2 (Remarks to the Author):

“Rhythmic evolution of laughter shows human vocal plasticity falls on a hominid continuum”
De Gregorio et al.

This study investigates the evolution of laughter's temporal structure by analyzing acoustic recordings from five hominid taxa: orangutans, gorillas, bonobos, chimpanzees, and humans. Using inter-onset intervals and rhythmic ratios, the authors report that laughter is isochronous across all great apes, a trait conserved from the last common ancestor. The study claims that while the fundamental rhythm (i.e., isochrony) is conserved, the tempo has accelerated and the variability of the rhythm has increased along the phylogenetic line toward humans. The authors conclude that these changes represent an "evolution of vocal control" and "rhythmic plasticity" that served as a precursor to speech. The manuscript presents an interesting comparative dataset, particularly in its attempt to quantify the temporal characteristics of laughter across hominids. However, there is a critical semantic and theoretical disconnect and between the results and the manuscript's title/framing. Since "isochrony" is conserved, the term "Rhythmic Evolution" in the title is misleading. What has evolved is not the rhythm itself, but the flexibility to modulate it. Furthermore, the mechanism driving this plasticity is likely rooted in respiratory control rather than purely vocal motor control.
Therefore, I think the manuscript is suitable for publication only if the following revisions are made:

1. Title & Framing: Change the title to reflect that rhythm is conserved while plasticity/flexibility evolved.
2. Mechanism: Explicitly discuss that the increased plasticity in humans is mechanically enabled by the shift from alternating (panting) to sustained egressive airflow (e.g., Davila Ross et al., 2009).
3. Limitations: Add a clear caveat regarding the small sample sizes (N=2–3) for non-human apes, noting that estimates of species-level variability (CV) are preliminary.
If the authors are unable to reframe the narrative from "Rhythmic Evolution" to "Evolution of respiratory control," or cannot address the mechanical definition of control, I recommend declining the submission to allow for transfer to a more specialized journal.

We thank the reviewer for their thoughtful feedback. We have revised the manuscript to clarify that while basic isochrony is largely conserved across great apes, tempo and rhythmic flexibility have evolved, and we now discuss the mechanical basis of human vocal plasticity. We have also added a brief caveat regarding the limited number of non-human ape individuals contributing to species-level variability estimates. Please see our point-by-point response for details.

The following are comments on the major points related to the above:

1. The Title and "Rhythmic Evolution"
The title "Rhythmic evolution... shows human vocal plasticity" is misleading because the study actually finds that the rhythmic category (isochrony) is conserved (did not evolve) and what changed was tempo and coefficient of variation (CV). The title implies a structural change in rhythm that the data does not support. A more accurate title should reflect the difference of tempo and variation across hominids.

Thank you for this thoughtful comment. We agree that our results show that a fundamental rhythmic property of laughter, namely isochrony, is largely conserved across great apes, while other temporal features, such as tempo and variability, change along the hominid lineage. In the original title, we used the term *rhythmic evolution* in a broad sense to refer to evolutionary changes in the temporal organization of laughter, including aspects such as tempo, regularity, and variability. However, we appreciate that this wording could be interpreted as implying that the rhythmic pattern itself evolved.

To avoid this potential ambiguity and better reflect the distinction you highlight, we have revised the title accordingly. The new title is: **Rhythm and timing in laughter reveal that human vocal plasticity falls on a hominid continuum.** We believe this revised formulation more clearly reflects the findings of the study while addressing your concern.

2. Misinterpretation of "Vocal Control" vs. "Respiratory Constraints"
The authors attribute the increase in rhythmic variability to "enhanced vocal control". However, the data and the previous literatures (e.g., Davila Ross et al., 2009; 2010) strongly suggest that this is actually a result of respiratory control.
For example, Davila Ross et al., (2009) reports that great ape laughter is predominantly "alternating egressive-ingressive" (panting). This means the "rhythm" of ape laughter is physically bound to the respiratory cycle. In contrast, humans produce "exclusively egressive" laughter, segmenting a single exhalation into multiple calls. Therefore, the observed "isochrony" in apes likely reflects the default output of the respiratory Central Pattern Generator (CPG). Actually, the authors themselves acknowledge in the manuscript that play disrupts the rhythm because it involves "compressing of the thorax" and disrupts the "respiratory cycle". So, the "evolution" observed is not necessarily the acquisition of vocal rhythmic ability, but rather, it is reasonable to think that the decoupling of vocalization from the strict 1:1 respiratory cycle. Humans show high variability because they can cognitively override the respiratory CPG, whereas apes are physiologically constrained by the panting mechanic. I think that the manuscript should reframe the discussion to address Respiratory Control/Flexibility rather than purely "Vocal Control."

We thank the reviewer for this thoughtful comment. We would like to clarify that the manuscript does not aim to contrast vocal control with respiratory control. Indeed, any modulation of vocal output necessarily involves modulation of the respiratory system, and we fully agree that the respiratory Central Pattern Generator (CPG) provides the physiological substrate underlying laughter production.

In the manuscript, we refer primarily to changes in the temporal organization of laughter (tempo, variability, and rhythm) rather than to “vocal control” per se. In fact, the expression “enhanced vocal control” appears only once, in the brief statement, and not in the main text, and we have and we have clarified the interpretative framework in the main text to provide additional context and avoid potential ambiguity.

We also acknowledge that the conserved isochrony observed in great apes likely reflects the default output of the respiratory CPG. However, the acceleration of laughter tempo and the increase in temporal variability observed along the hominid lineage indicate additional modulation beyond this baseline mechanism. These changes likely involve enhanced cortical or voluntary modulation of vocal production, allowing greater temporal flexibility. Importantly, our analyses further show that only humans modulate laughter tempo according to social context, producing faster laughter during tickling than during play, whereas such context-sensitive modulation is absent in the other great apes.

Finally, the scenario suggested by the reviewer—namely a progressive loosening of the strict coupling between vocal output and the respiratory cycle—appears broadly

compatible with our interpretation of increased temporal flexibility in laughter along the hominid lineage. To acknowledge this perspective, we have now added a brief sentence noting that these evolutionary changes may relate to the progressive loosening of the coupling between vocal output and the respiratory cycle described in great ape laughter (e.g., Davila-Ross et al., 2009), the section now reads (Line 64): Tickling laughter was highly regular, while play laughter deviated significantly from strict regularity. This difference likely reflects the dynamic nature of social play, which involves the torsion, tensioning, thumping, and compressing of the thorax (Pellis et al., 2010; Palagi et al. 2016), and with it, the disruption of a would-be regular respiratory cycle (Bramble & Carrier, 1983). The "unaltered" rhythm of tickling laughter offers, thus, a stable window onto the evolutionary changes undergone by the phonatory-respiratory system of hominids. These changes may also relate to the progressive loosening of the coupling between vocal output and the respiratory cycle described in great ape laughter (e.g., Davila-Ross et al., 2009)."

3. Inadequacy of Sample Size for Evolutionary Claims
The sample sizes are insufficient to draw robust conclusions about species-specific evolutionary trends, particularly regarding "variability" and "plasticity," which are highly subject to individual differences. While the authors use GLMMs to account for individual identity , the statistical power relies heavily on the number of calls rather than the number of independent biological units (individuals). The claim that variability decreases with phylogenetic distance is weak when the datapoints representing "distance" are based on so few individuals.

We thank the reviewer for raising this concern. We would like to clarify that our analyses explicitly account for individual identity using GLMMs, so that each individual's contribution is properly modeled and calls are not treated as fully independent biological units. Our dataset includes 17 individuals, which is comparable to previous work such as Davila-Ross et al. (2009), who analyzed 22 individuals across one additional primate species (siamangs) and 892 individual calls. Importantly, for our rhythmic analyses, only bouts containing at least two calls were used to compute *tk* values, and at least three calls to compute *rk* values (since *rk* requires two *tk* intervals). This ensures that our dataset is fully congruous and statistically appropriate for estimating species-level trends in temporal variability and rhythmic plasticity, while properly controlling for individual differences. We further note that our dataset includes a substantial number of temporally-resolved datapoints: 458 *tk* values and 316 *rk* values.

Regarding our estimates of CV, we have now added a brief clarification in the manuscript noting that the number of individuals per non-human ape species in the present dataset is limited, and that future studies with larger samples will help further characterize

species-level estimates of variability. It now reads (line 82): “Phylogenetically-informed statistical analyses reveal a gradual shift toward greater temporal variability in laughter (LMM4, $p = 0.004$, Tab. SM3), with humans exhibiting the highest variability in laughter tempo (Tab. SM4), a result consistent with enhanced rhythmic range, a hallmark of advanced vocal control. Although this pattern emerges across the present dataset, the number of individuals per species remains limited, and future work with larger samples will help further refine species-level estimates of variability.”

4. Methodological Concerns: Measuring Onsets in "Noisy" Signals
The validity of the rhythm analysis depends entirely on the precise measurement of the call onset. Davila Ross et al. (2009) describes ape laughter as "overwhelmingly irregular and noisy". Unlike the clear, voiced harmonics of human laughter, ape laughter is often a breathy pant. Indeed, the Davila Ross et al. (2009) (particularly concerning orangutan tickling) also includes spectrograms where the onset is difficult to identify. Therefore, the author should describe the method to demonstrate that the onset of the analyzed vocalization can be accurately detected.

We thank the reviewer for raising this point. We would like to be clear that all call onsets were measured with great care, using high-quality recordings and rigorous spectrographic inspection. The sounds were analyzed using FFT-based narrowband and wideband spectrograms (40-ms and 8-ms Hanning windows), as well as spectral slices and waveforms; the latter allowed us to systematically identify sound onsets and offsets. Our methodology ensures precise segmentation of all vocalizations, and we are confident that the measurements presented are reliable. We would also like to clarify that there was no intention to misrepresent or inaccurately measure the data.

Reviewer #3 (Remarks to the Author):

De Gregorio and colleagues have prepared a very interesting short manuscript on their analysis of the rhythmic properties of laughter across several great ape species. I found the paper to be well written and of sufficient relevance and potential impact for the journal's general readership.

We thank the reviewer for these encouraging comments and for recognizing the relevance and potential impact of our study. We greatly appreciate the positive feedback on the clarity and presentation of the manuscript.

I have just two small comments about the methods on which I would appreciate further explanation/consideration by the authors:

- The use of infant humans is a good idea, not only in matching the age range of the existing sample but also because older human children exhibit forms of volitional laughter reflective of emergent social strategies (e.g. echoing or mimicking adults: the example of when small children laugh in chorus with adults without knowing the reason for the laughter). In the sample reported in the paper, I however see that one individual is 36 months old and was wondering if their behaviours may be more reflective of these kinds of social developments than the substantially younger infants (11, 12, and 15mo). Can the authors justify the inclusion of this child as developmentally equivalent to the other 3 individuals?

We thank the reviewer for this insightful comment. The inclusion of the 36-month-old child was based on their overall developmental stage in relation to the vocal and motor abilities relevant for laughter production. While older than the other infants (11–15 months), this child still displayed spontaneous laughter in response to tickling, comparable to the younger individuals, and there was no indication that this laughter was produced volitionally. We carefully verified that the recorded laughter bouts reflected reflexive, play-induced responses rather than socially mediated or volitional forms of laughter, ensuring developmental equivalence for the purposes of our analyses.

- I noticed that the individual identity was modelled as a random effect for some, but not all of the analyses. Can the authors please explain their decision-making around this? In the above case, for example, the 36 month old could be showing more sophisticated/complex behaviours compared to the younger individuals that could play a role in driving inter-species effects.

We thank the reviewer for raising this important point. When structuring the analyses, we initially attempted to include individual identity as a random effect across all models considering tk duration (log_dur). However, in the model testing *tk* as a function of species in interaction with context, including individual identity, resulted in a rank-deficient fit. For this reason, we used the recording file ID (from which the *tk* values were extracted) as a random effect in that model. To maintain consistency across this specific series of models, we initially adopted the same random-effect structure in the other analyses as well.

The reviewer's point is nevertheless valid. We verified that including individual identity as a random effect does not substantially change the results (it only leads to minor adjustments in the decimal values of the p-values, see new version of the supplement information). In light of this comment, we have now revised the analyses and present tk models including individual identity as a random effect wherever possible. Only in the case of the model affected by the rank-deficiency warning do we retain file ID as the random effect. The results do not change.

In Figure 1, I would recommend making more distinct the on- vs off-ratio ranges (e.g. using different colours) and giving some explanation of those in the figure legend so that they can be understood without reading the Methods.

Thank you for the suggestion. We agree that making the on- vs off-ratio ranges more visually distinct will improve the clarity of the figure. We have revised Figure 1 accordingly and added a brief explanation in the figure legend so that the ranges can be understood without referring to the Methods: “**A)** Probability density function of rhythm ratios (rk) in the two behavioral contexts (play, in yellow, and tickling in green) derived from 140 laughter bouts across 17 individuals. White lines highlight on-integer (0.440 < rk < 0.555,, lighter shade) and off-integer (0.400 < rk < 0.440 and 0.555 < rk < 0.600, darker shade) ratio ranges. *Denotes p<0.05, a statistically significant match between the empirical distribution and a small integer ratio rhythmic category.”

Here is the new version of the figure:

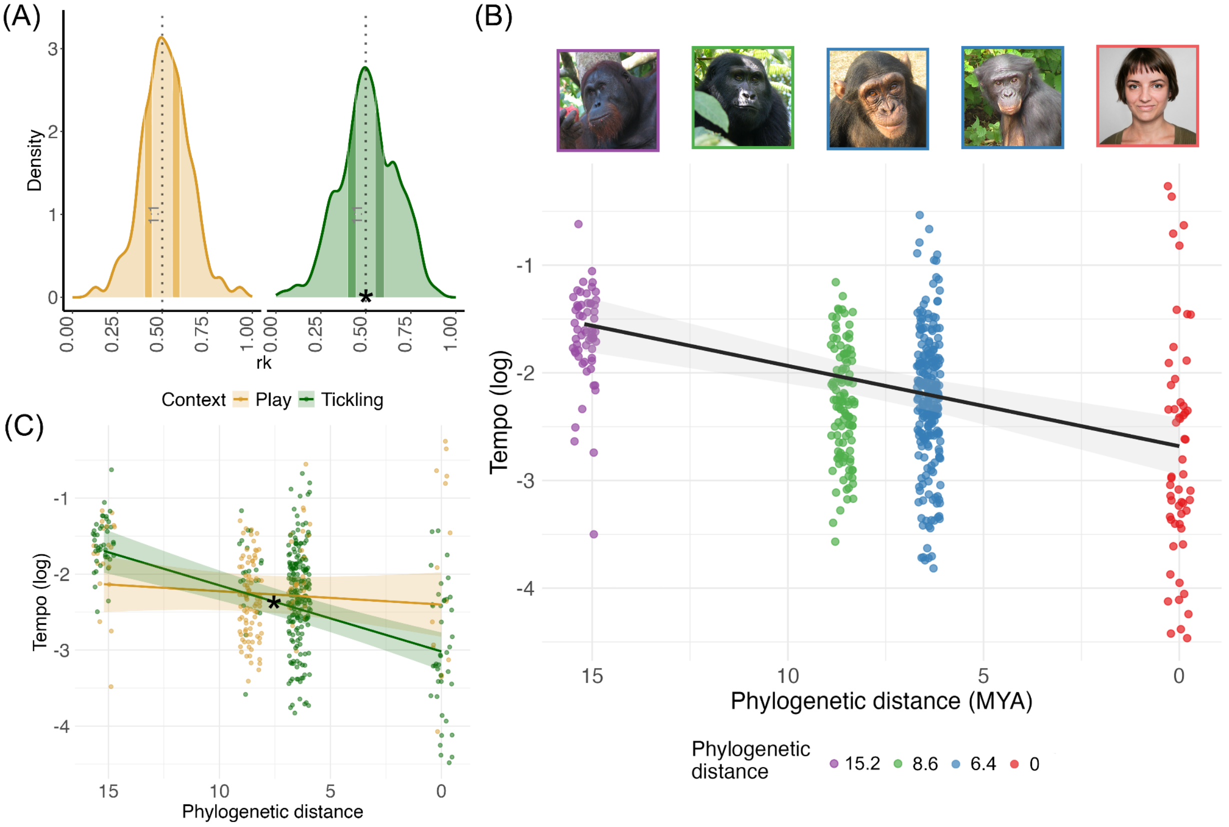

Authors’ response - COMMSBIO-25-11179A

I have reviewed the responses to reviewer #2. In my professional opinion, the changes made to most of the points are reasonable, and the authors have even changed the title. The methodological concerns raised by reviewer #2 are valid, particularly regarding sample sizes. This challenge is common in my field, but the authors appropriately employ the necessary frequentist statistical methods to address it.

The only feedback that the authors did not adequately address in their revisions & responses was point #4, which pertains to the 'noisiness' of the signals. I believe the authors should acknowledge the presence of 'noisiness' in their manuscript and explain how each protocol helps minimize the confounding factors present in these recordings.

We would like to thank the reviewer for the positive and constructive feedback on our revised manuscript. We are very pleased that the changes made, including the modification of the title and the revisions addressing the reviewers’ points, are considered appropriate.

Regarding point #4, concerning the “noisiness” of the signals, we agree that this is an important aspect to clarify in the manuscript. We have therefore revised the text to better acknowledge and describe how noise was handled in the dataset. Specifically, we now state (lines xx):

“Recordings with a signal-to-noise difference below 2 dB were discarded to increase the quality of the recordings. If noise masked the recordings with random fluctuations or if the onset/offset of the calls were unclear, these recordings were not included in the study. While it needs to be noted that the recordings were not perfectly clean from noise, which is to be expected from the majority of such acoustic studies, the impact of such factors is smaller when the focus is on temporal patterns, as in this study. Start times were aligned to 0.0 s and direct current offsets introduced by tape recording were corrected to center the waveform on the zero-voltage axis and to remove low-frequency recording artifacts.”

We hope that this revision adequately addresses the concern raised.
